# Joachim Küchenhoff, Martin Teising: Sich selbst töten mit Hilfe Anderer. Kritische Perspektiven auf den assistierten Suizid

**DOI:** 10.3205/zma001622

**Published:** 2023-06-15

**Authors:** Luise Wagner

**Affiliations:** 1 University Hospital Jena, Institute of General Medicine, Jena, Germany

## Bibliographical details

Joachim Küchenhoff, Martin Teising

**Sich selbst töten mit Hilfe Anderer. Kritische Perspektiven auf den assistierten Suizid**

Publisher: Psychosozial-Verlag

Year of publication: 2022, 275 pages, price: € 34,90

ISBN: 978-3-8379-3171-6

## Review

In 2020, the German Federal Constitutional Court deemed the prohibition of businesslike assisted suicide unconstitutional. In doing so, it made a judgment of historic singularity. Its reasoning echoes the increasingly individualistic orientation of both individual and social life. Two years later, Joachim Küchenhoff and Martin Teising published a book that critically examines this verdict and its significance for the individual and the “others” considered in the title from different perspectives.

A contextualizing preface by the editors is followed by a total of 14 separate contributions in four parts. The most comprehensive one is the first part, which deals with the framework of the discussion on assisted suicide. Here, the focus is particularly on the addressed verdict and its “misinterpreted” understanding of the concepts of autonomy and freedom as a central point of criticism. This is followed by reflections on assisted suicide in medicine in the second part. In addition to a broad plea by physician and philosopher Giovanni Maio to society in general and medicine in particular for more commitment to not give people a reason to consider suicide, the other two contributions focus primarily on the psychiatric context. The third part addresses the relationship between suicidal persons and their helpers, which has been little discussed in the public debate so far. This is done primarily from a psychoanalytic perspective, which repeatedly traces the psychodynamic process to the root of suicidality. Finally, the fourth part with its last two contributions is devoted to social and cultural aspects of assisted suicide. Noteworthily, the contribution by Lisa Werthmann-Resch, in which she analyzes the dynamics of suicide in “Winterreise” by Franz Schubert and in the contemporary same-titled film by Hans Steinbichler, stands out due to its unique approach.

The broadness of perspectives as well as the resulting solutions and demands (in the sense of a more or less constructive criticism) vary between the contributions from broad and general to focused and concrete: powerful philosophical argumentations stimulate far-reaching thoughts, but may leave solution-oriented readers unsatisfied due to the lack of a practicable outlook. In other contributions, the discussed aspects and concrete possibilities of dealing with them are vividly illustrated by means of case reports rooted in history or the authors‘ own experiences.

The cover blurb promises a broad interdisciplinary approach to the topic. However, at first glance, the 17 authors appear to be quite homogeneous due to their mostly psychiatric and psychotherapeutic, especially psychoanalytic backgrounds. This fact is also mentioned in the preface of the editors. Indeed, redundancies of some central aspects in the various contributions cannot be denied. For instance, given the psychoanalytic focus it is not surprising that Freud appears regularly in the contributions, both as the forefather of psychoanalytic thought as well as a prominent historical example of a person dying with the help of others. Furthermore, multiple contributions elaborate on the paradox of two incompatible aspirations: autonomy understood as absolute independence from others on the one hand, and on the other hand the fundamentally social *conditio humana* resulting in a lifelong dependency on others. Consistently, the repeated emphasis on this conflict is true to the title of the book, which pays particular attention to those others: the suicide assistants, therapists, relatives, and society.

Despite the clear psychoanalytical emphasis, the book offers interested readers a variety of approaches and lines of argumentation to engage themselves in selected aspects of assisted suicide. This diversity of reflections on such an existential topic evokes in the readers different degrees of feeling addressed and being able to comprehend the individual contributions. Thus, the book can provoke in them what it has also demanded of its authors: a critical examination of several points of discussion surrounding assisted suicide and the search for one's own inner attitude towards it. Sentiments of approval or disapproval induced during the reading can provide helpful clues on the way to one's own position.

However, the reader needs to pay close attention to terminology, since in some contributions the distinction between assisted suicide and voluntary euthanasia is not consistent. Also, sudden jumps in the discussion between different years, nations and thus also different legal and social frameworks can quickly confuse, especially when dealing with the topic for the first time. Here, consistent definitions of terms across the contributions and explicit declarations of temporal and geographical boundaries would have been desirable for the benefit of clarity.

Therefore, the book is rather less suitable as a general overview or introductory reading on the discussion about assisted suicide. Practitioners, educators, students, and other interested parties who wish to (further) explore the topic of assisted suicide, fundamental questions of life and death, and the role of physicians and therapists in particular, can find stimulating thoughts and impulses for reflection and discussion in the book. An interest in psychoanalysis and at least a rudimentary understanding of its theoretical principles are advantageous.

## Competing interests

The author declares that she has no competing interests.

